# Impaired PGE_2_-stimulated Cl^-^ and HCO_3_^-^ secretion contributes to cystic fibrosis airway disease

**DOI:** 10.1371/journal.pone.0189894

**Published:** 2017-12-27

**Authors:** Zachary M. Sellers, Beate Illek, Miriam Frankenthal Figueira, Gopika Hari, Nam Soo Joo, Eric Sibley, Jackson Souza-Menezes, Marcelo M. Morales, Horst Fischer, Jeffrey J. Wine

**Affiliations:** 1 Division of Pediatric Gastroenterology, Hepatolfifogy, and Nutrition, Stanford University, Palo Alto, CA, United States of America; 2 Cystic Fibrosis Research Laboratory, Stanford University, Palo Alto, CA, United States of America; 3 Children’s Hospital Oakland Research Institute, Oakland, CA, United States of America; 4 Carlos Chagas Filho Biophysics Institute, Federal University of Rio de Janeiro, Rio de Janeiro, RJ, Brazil; 5 Core for Ecology and Socio Environmental Development, Federal University of Rio de Janeiro, Macaé, RJ, Brazil; University of Alabama at Birmingham, UNITED STATES

## Abstract

**Background:**

Airway mucociliary clearance (MCC) is an important defense mechanism against pulmonary infections and is compromised in cystic fibrosis (CF). Cl^-^ and HCO_3_^-^ epithelial transport are integral to MCC. During pulmonary infections prostaglandin E_2_ (PGE_2_) production is abundant.

**Aim:**

To determine the effect of PGE_2_ on airway Cl^-^ and HCO_3_^-^ secretion and MCC in normal and CF airways.

**Methods:**

We examined PGE_2_ stimulated MCC, Cl^-^ and HCO_3_^-^ secretion using ferret trachea, human bronchial epithelial cell cultures (CFBE41o- with wildtype CFTR (CFBE41 WT) or homozygous F508del CFTR (CFBE41 CF) and human normal bronchial submucosal gland cell line (Calu-3) in Ussing chambers with or without pH-stat.

**Results:**

PGE_2_ stimulated MCC in a dose-dependent manner and was partially impaired by CFTR_inh_-172. PGE_2_-stimulated Cl^-^ current in ferret trachea was partially inhibited by CFTR_inh_-172, with niflumic acid eliminating the residual current. CFBE41 WT cell monolayers produced a robust Cl^-^ and HCO_3_^-^ secretory response to PGE_2_, both of which were completely inhibited by CFTR_inh_-172. CFBE41 CF cells exhibited no response to PGE_2_. In Calu-3 cells, PGE_2_ stimulated Cl^-^ and HCO_3_^-^ secretion. Cl^-^ secretion was partially inhibited by CFTR_inh_-172, with additional inhibition by niflumic acid. HCO_3_^-^ secretion was completely inhibited by CFTR_inh_-172.

**Conclusions:**

PGE_2_ stimulates bronchotracheal MCC and this response is decreased in CF. In CF airway, PGE_2_-stimulated Cl^-^ and HCO_3_^-^ conductance is impaired and may contribute to decreased MCC. There remains a CFTR-independent Cl^-^ current in submucosal glands, which if exploited, could represent a means of improving airway Cl^-^ secretion and MCC in CF.

## Introduction

Cystic fibrosis, caused by mutations in the cystic fibrosis transmembrane conductance regulator (CFTR), is characterized by defective Cl^-^ and HCO_3_^-^ epithelial ion transport. In the airways this results in thick, sticky mucus, impairing airway surface liquid (ASL) height and mucociliary clearance (MCC). In healthy individuals, routine microbial insults of the lung are cleared through a non-pathologic inflammatory response, coupled with bronchotracheal MCC of mucus-trapped pathogens, thereby preventing obstruction and infection [1]. In cystic fibrosis (CF), defective MCC leads to bronchiectasis, chronic infections, and progressive loss of lung function. Bronchotracheal Cl^-^ and HCO_3_^-^ secretion contribute to ASL height and MCC through effects on extracellular hydration and mucin expansion [2–4]. In the model put forth by Haq *et al*., defective Cl^-^ and HCO_3_^-^ transport in CF leads to a dehydrated and acidic ASL. Dysregulation of the epithelial Na^+^ channel (ENaC) causes Na^+^ hyperabsorption, further dehydrating the ASL layer. Water moves out of the mucus layer and eventually out of the periciliary layer, which coupled with increases mucus viscosity due to the acidic environment, results in a thick, viscous layer that compresses the cilia and impairs MCC [5].

Airway anion secretion occurs in response to microbial infection [6] and inflammatory mediators. In infected airways, prostaglandin E_2_ (PGE_2_) is abundantly produced by epithelia and infiltrating inflammatory cells, and is found in bronchioalveolar lavage fluid, sputum, and airway epithelium [7–10]. During acute CF pulmonary exacerbations, sputum PGE_2_ levels can increase over four-fold [9]. In the intestines, PGE_2_ stimulates Cl^-^, HCO_3_^-^, and mucin secretion *via* cAMP, Ca^2+^, and PI3K (phosphatidylinositol 3-kinase) signaling [11, 12]. In the duodenum CFTR is an important HCO_3_^-^ exit pathway for PGE_2_-stimulated HCO_3_^-^ secretion, but unlike many other stimuli, PGE_2_ may also stimulate HCO_3_^-^ secretion through CFTR-independent exit pathways [13, 14]. In the airways, PGE_2_ has been shown to increase iodide transport and short-circuit current (*I*_*sc*_), which has led to a presupposition that PGE_2_ stimulates anion transport through CFTR [15–17], however, its specific role in Cl^-^ and HCO_3_^-^ secretion in CF airways remains unclear.

We hypothesized that PGE_2_ signaling plays an important role in the normal response to airway insult by activating, *via* CFTR, Cl^-^ and HCO_3_^-^ dependent fluid secretion that optimizes mucus clearance, and that in CF, defective PGE_2_-stimulated anion secretion contributes to CF airway disease. In order to specifically study Cl^-^ *vs*. HCO_3_^-^ transport, we crafted a series of experiments that promoted preferential transport of Cl^-^ *vs*. HCO_3_^-^, performed ion substitution studies, and used pH-stat titration for measurement of HCO_3_^-^ secretion. We studied this process in cell culture models of bronchial surface epithelial cells, submucosal glandular cells, and intact trachea to determine the effects of PGE_2_ on Cl^-^ and HCO_3_^-^ secretion in distinct components of the airway, and assessed how these components may contribute to MCC.

## Materials and methods

### Cell culture and tissues

16HBE14o-, CFBE41o- + wildtype CFTR (CFBE41 WT), CFBE41o- + homozygous F508del CFTR (CFBE41 CF), and Calu-3 cell lines were cultured using procedures similar to previously, according to standard protocols [18–20]. Primary cultures from human bronchial epithelial cells and CF nasal polyp explant epithelial cells were obtained from Dr. Walter Finkbeiner (University of California, San Francisco) and were cultured using published protocols [21, 22]. Calu-3 cells were purchased from ATCC (Manassas, VA). All cells were grown at air-liquid interface and used when transepithelial resistance indicated intact monolayer. Calu-3 cells were used at about 300Ω.cm^2^ and bronchial epithelial cell lines at about 1000Ω.cm^2^. *Mustela putorius* ferrets of 6–36 months old were obtained 1–2 hours postmortem by pentobarbital sodium injection and tissues were transported in ice-cold PhysioSol^TM^ (Hospira, IL) solution. Trachea was obtained from just below the larynx to just above the carina. Tissues were transferred to ice-cold Krebs Ringer HCO_3_^-^-buffered solution and gassed with 95% O_2_/5% CO_2_ until used, usually within 6 hours of procurement [2]. All protocols for handling animal tissues at Stanford were approved by the Administrative Panel on Laboratory Animal Care (Stanford’s Institutional Animal Care and Use Committee: IACUC protocol#: 10048).

### Mucociliary clearance

Experiments were performed in a manner similar to that done previously [2]. The dorsal muscular portion of the trachea was cut along its entire length and the opened trachea with cartilage intact was pinned mucosal side up in a chamber allowing the serosal side to be bathed in a 37°C Kreb’s Ringer HCO_3_^-^-buffered solution with indomethacin (1 μM). The mucosal side was exposed to warm, humidified air (95% O_2_/5% CO_2_). Mounted trachea was stabilized in the chamber for 15 minutes, except when pretreated with CFTR_inh_-172 inhibitor, and then the bath was discarded and replaced with fresh solution. For CFTR inhibitor studies, the trachea was bathed bilaterally with CFTR_inh_-172 (20 μM) for 30 minutes and then CFTR_inh_-172 remained in the serosal bath for the entire experiment. Xerox ink particles were deposited at the proximal portion of the trachea and a video camera captured images every 20 seconds, tracking the particles as they moved towards the distal end. Measurements (mm/min) were averaged over 5 minutes and tracked for 30 minutes. Tissue viability was tested at the end of each experiment with forskolin (10 μM) and carbachol (0.3 μM).

### Measurement of *I*_*sc*_

Snapwell inserts with confluent cell culture monolayers were mounted in an Ussing chamber (Physiologic Instruments P2300), and transepithelial voltage was clamped to zero millivolts using a voltage clamp meter (Physiologic Instruments VCC600), and *I*_*sc*_ recorded on a computer using data acquisition software (LabChart 8, ADInstruments). To monitor changes in transepithelial resistance, a voltage pulse of 1 mV was applied every 60 seconds with measurement of resultant deflections of *I*_*sc*_. Ohm’s Law was used to calculate transepithelial resistance. Ussing chambers were kept at 37°C with a temperature-controlled water bath circulator and both mucosal and serosal solutions were continuously gassed with 95% O_2_/5% CO_2_. For HCO_3_^-^-free experiments mucosal and serosal solutions were gassed with 100% O_2_. All snapwells were rinsed in unbuffered HCO_3_^-^-free solution prior to placement in Ussing chambers. For tracheal tissues, the tissue was placed in ice-cold PhysioSol^TM^ (Hospira, IL) solution until further dissection, at which time it was placed in solution containing indomethacin (10 μM) to inhibit endogenous prostaglandin release due to dissection trauma. The tracheal submucosal layer containing cartilage was left intact, however, the outer layer covering the cartilage was bluntly dissected under a dissecting microscope with transillumination to ensure no over dissection. Tissue was bathed in indomethacin-containing solution during the entire dissection. Tissues were secured in sliders with steel pins, which are located sufficient distance away from the aperture so as not to interfere with ion transport measurements. Indomethacin (10 μM, bilaterally) was present during Ussing chamber experiments to prevent *de novo* formation of prostaglandins. Amiloride (10 μM, mucosal), to inhibit epithelial Na^+^ channel (ENaC), was added at the beginning of the experiment and was present throughout the entire experiment. For CFTR_inh_-172 pre-treatment, CFTR_inh_-172 (20 μM, mucosal) was added at least 30 minutes prior to PGE_2_ stimulation. For HCO_3_^-^-free experiments, acetazolamide (300 μM, bilateral) was used to inhibit carbonic anhydrase, in addition to O_2_ gassing and HCO_3_^-^ removal from solutions.

### Measurement of HCO_3_^-^ secretion by pH-stat

The pH-stat method, which measures the amount of HCl needed to keep the luminal bath at a constant pH using a pH electrode, was used to measure HCO_3_^-^ secretion. Automatic titrators (Metrohm Titrando 902) were used to titrate 0.2 μL aliquots of 5 mM HCl into the mucosal bath at a steady rate in order to keep from under- or overshooting the set pH. The pH was set to 6.9 in order to prevent activation of apical HCVN1 proton channels which are activated at pH >7.0 [23]. Tiamo software (Metrohm) was used to control the rate of titration and continuously measure the amount titrated and pH. Bicarbonate secretory rates (μmol.cm^2^.h^-1^) were calculated in 5 minute intervals by noting the amount titrated, the concentration of titrant, and the surface area of the slider aperture. Short-circuit measurements were simultaneously performed during pH-stat measurements in a similar manner as Cl^-^ secretion measurements, with a few exceptions. First, cell monolayers were not voltage pulsed. To monitor transepithelial resistance, the voltage clamp was released and the open circuit voltage was recorded every 10 minutes. During this time the auto-titrator was briefly paused to ensure no interference. Second, the serosal solution was bathed with 95% O_2_/5% CO_2_ (similar to Cl^-^ experiments), but the mucosal solution was bathed with 100% O_2_ to prevent base formation from carbonhic anhydrase conversion of CO_2_.

### Solutions

The Krebs Ringer HCO_3_^-^-buffered solution for MCC consisted of (in mM): NaCl 115, K_2_HPO_4_ 2.4, KH_2_PO_4_ 0.4, NaHCO_3_ 25, MgCl_2_ 1.2, CaCl_2_ 1.2, Glucose 10. Solutions for tracheal Ussing chamber experiments consisted of the following in mM. Mucosal: NaGluconate 115, K_2_HPO_4_ 2.4, KH_2_PO_4_ 0.4, NaHCO_3_ 25, Mg(Gluconate)_2_ 1.2, Ca(Gluconate)_2_ 4, Mannitol 10; Serosal: NaCl 115, K_2_HPO_4_ 2.4, KH_2_PO_4_ 0.4, NaHCO_3_ 25, MgCl_2_ 1.2, CaCl_2_ 1.2, Glucose 10. For Cl^-^ secretion experiments with human bronchial epithelial cells and Calu-3 cells, solutions were as following in mM: Mucosal: NaGluconate 120, NaHCO_3_ 25, KH_2_PO_4_ 3.3, K_2_HPO_4_ 0.8, Ca(Gluconate)_2_ 4, Mg(Gluconate)_2_ 1.2, Mannitol 10; Serosal: NaCl 120, NaHCO_3_ 25, KH_2_PO_4_ 3.3, K_2_HPO_4_ 0.8, CaCl_2_ 1.2, MgCl_2_ 1.2, Glucose 10. For HCO_3_^-^ secretion measurements by *I*_*sc*_ only, solutions were similar to the above, except (in mM): Mucosal: NaCl 120, NaHEPES 25. For pH-stat experiments, solutions were as follows (in mM): Mucosal: NaCl 115, NaGluconate 25, KCl 5, CaCl_2_ 1.2, MgCl_2_ 1.2, Mannitol 10; Serosal: NaCl 120, NaHCO_3_ 25, KH_2_PO_4_ 3.3, K_2_HPO_4_ 0.8, CaCl_2_ 1.2, MgCl_2_ 1.2, Glucose 10. All solutions had an osmolarity of approximately 290 mOsm, as determined by a vapor pressure osmometer (Wescor, 5500).

### Inhibitors

As stated above, CFTR_inh_-172 (20 μM, mucosal) was used to inhibit CFTR [24], amiloride (10 μM, mucosal) to inhibit ENaC, acetazolamide (300 μM, bilaterally) to inhibit carbonic anhydrase, and indomethacin (10 μM, bilaterally) to inhibit prostaglandin formation *via* cycloxygenase. Additionally, bumetanide (10 μM, serosal) was used to inhibit the basolateral Na^+^:K^+^/2Cl^-^ (NKCC) channel and niflumic acid (100 μM, mucosal) to inhibit Ca^2+^-activated Cl^-^ channels. Oubain (10 μM, mucosal) was used to inhibit apical non-gastric H^+^/K^+^ ATPase. All drugs (inhibitors plus PGE_2_, forskolin, carbachol, adenosine-triphosphate (ATP)) were obtained from Sigma-Aldrich.

### Statistical analysis

Mean ± standard error of the mean (SEM) were calculated for all experiments with at least three replicates. Statistical significance between groups was determined using paired and unpaired Student’s t-test, as appropriate. Time course comparisons were performed using one-way analysis of variance (ANOVA). Significance was determined at P values < 0.05.

## Results

### Mucociliary clearance

**Fig 1:**
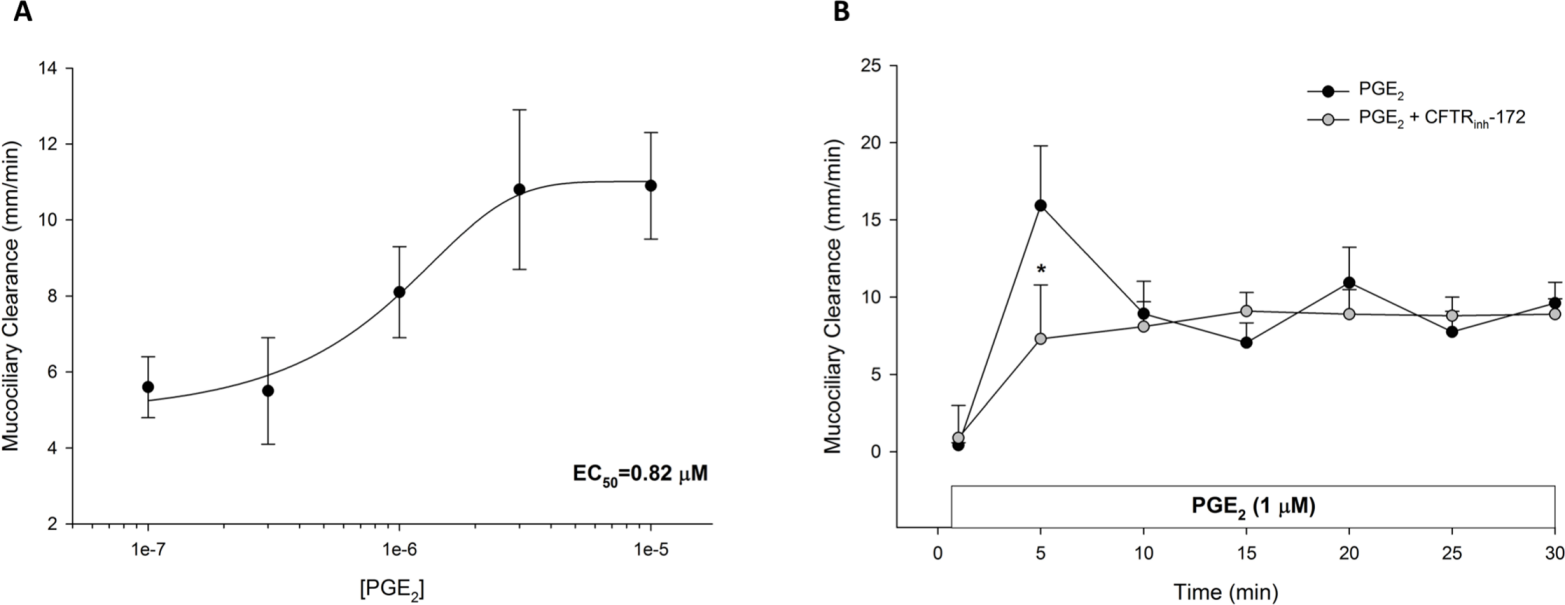
PGE_2_-stimulated mucociliary transport in ferret trachea. **A.** PGE_2_ stimulates a dose-dependent increase in MCC in ferret trachea. Each tissue was exposed to 2–3 doses of PGE_2_ for 30 minutes each (n = 3 each dose). Data are shown as the mean PGE_2_-stimulated increase in MCC over baseline ± SEM. The half-maximal effective concentration (EC_50_) is noted in lower right corner. **B.** Timecourse of PGE_2_-stimulated MCC with and without CFTR inhibition (n ≥ 6 each). For CFTR inhibition, tissues were bathed in apical and serosal solution for 30 minutes with CFTR_inh_-172 (20 μM) prior to the 15-minute period and kept in the serosal bath for the length of the experiment. PGE_2_ (1 μM) was added to the serosal bath. Circles represent means with bars indicating SEM. Asterisks represent P < 0.05 by ANOVA.

We first examined the effect of PGE_2_ on MCC, with a validated model of MCC using ferret trachea *ex vivo* [2]. Serosal exposure of PGE_2_ in concentrations ranging from 10^−7^ M to 10^−5^ M (n = 3 each dose), produced a dose-dependent increase in MCC with an EC_50_ of 0.82 μM (Fig 1A). In examining the timecourse of stimulation, PGE_2_ (1 μM, serosal) increased MCC with an initial peak at 5 minutes, followed by a lower sustained response (n ≥ 6 each). Pre-treatment with CFTR_inh_-172 (20 μM; n ≥ 6 each) attenuated the initial peak (P < 0.05), but did not affect the sustained plateau (Fig 1B). These data suggest that PGE_2_-stimulated MCC is partially CFTR-dependent, and may contain a CFTR-independent mechanism for clearance.

### Cl^-^ secretion

**Fig 2:**
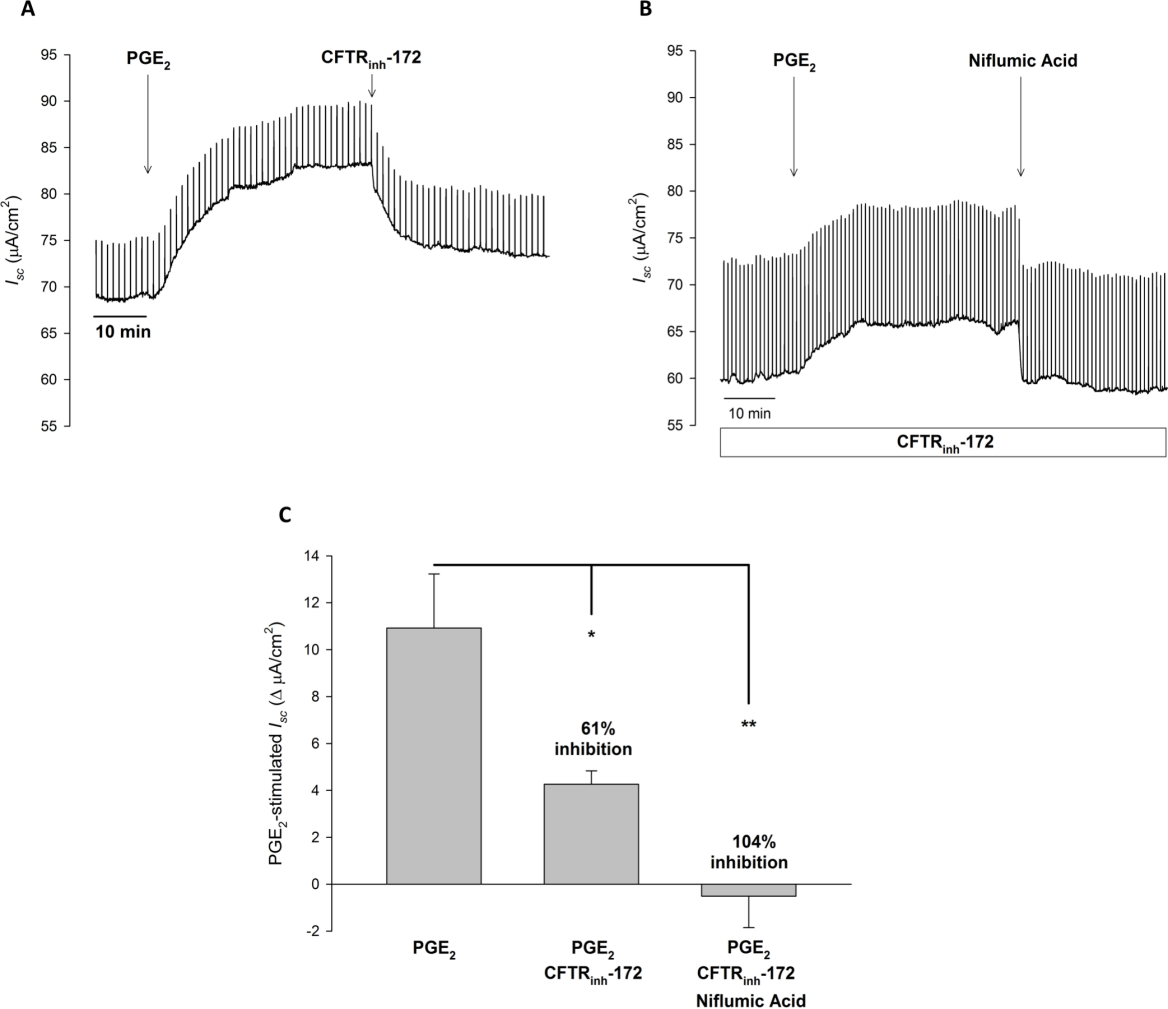
In ferret trachea, PGE_2_ stimulated *I*_*sc*_ is mediated by CFTR and Ca^2+^-activated Cl^-^ channels. **A.** Representative *I*_*sc*_ trace with vertical deflections indicating the change in *I*_*sc*_ after a 1 mV pulse was applied (every 1 minute). Ferret trachea was exposed to serosal to mucosal Cl^-^ gradient with equivalent bilateral HCO_3_^-^. PGE_2_ (1 μM, serosal) was added to ferret trachea after a baseline period of ≥ 10 minutes, with CFTR_inh_-172 (20 μM, mucosal) added after 30 minutes. **B.** Representative *I*_*sc*_ trace of ferret trachea incubated in CFTR_inh_-172 (20 μM, mucosal) for at least 30 minutes prior to PGE_2_ (1 μM, serosal) stimulation. After 30 minutes, niflumic acid (100 μM, mucosal) was added. **C.** Change in PGE_2_-stimulated *I*_*sc*_ (mean ± SEM, n ≥ 5) in ferret trachea, with comparisons between no inhibition, CFTR inhibition, or CFTR and Ca^2+^-activated Cl^-^ inhibition. Asterisks denote significance by Student’s t-test (*, P < 0.05, **, P < 0.01). Mean percent inhibition compared to PGE_2_ stimulation alone noted.

Ferret tracheal MCC has been shown to be highly dependent on transepithelial Cl^-^ transport [2]. Thus, to correlate PGE_2_-stimulated MCC rate to Cl^-^ transport, we examined PGE_2_-stimulated *I*_*sc*_ with ferret trachea mounted in Ussing chambers with a serosal to mucosal Cl^-^ gradient. As seen in Fig 2A, in the presence of amiloride (10 μM, mucosal), PGE_2_ (1 μM, serosal) stimulated a significant increase in *I*_*sc*_ over baseline (65.83 ± 12.01 *vs*. 78.61 ± 14.43 μA/cm^2^, P < 0.01, n = 7). Subsequent addition of CFTR_inh_-172 (20 μM, mucosal) caused a significant, but not complete, inhibition of PGE_2_-stimulated *I*_*sc*_ (PGE_2_: 76.75 ± 14.24 *vs*. CFTR_inh_-172: 69.90 ± 13.82 μA/cm^2^, P < 0.01, n = 7) (Fig 2A and 2C). Further addition of bumetanide (10 μM, serosal) to block basolateral Cl^-^ uptake *via* NKCC, completely abolished the remaining PGE_2_-stimulated *I*_*sc*_ (-10.23 ± 4.33 ΔμA/cm^2^ from baseline, n = 7). These results show that: 1) PGE_2_-stimulated *I*_*sc*_ is reflective of transepithelial Cl^-^ secretion, and 2) CFTR is responsible for the majority, but not all, of PGE_2_-stimulated Cl^-^ secretion. With the ability of PGE_2_ to stimulate cAMP and Ca^2+^ intracellular signaling pathways, we next examined if the remaining bumetanide-sensitive *I*_*sc*_ was from activation of Ca^2+^-activated Cl^-^ channels. In similar experiments, we examined the ability of niflumic acid (NFA: 100 μM, mucosal), a Ca^2+^-activated Cl^-^ channel inhibitor, to inhibit CFTR-independent *I*_*sc*_. In these experiments tissues were pre-treated with amiloride (10 μM, mucosal) and CFTR_inh_-172 (20 μM, mucosal) for at least 30 minutes prior to PGE_2_ stimulation. Fig 2B and 2C show that NFA eliminates PGE_2_-stimulated *I*_*sc*_ in the presence of CFTR_inh_-172 (Baseline: 62.93 ± 8.58 *vs*. NFA: 60.22 ± 8.11 μA/cm^2^, n = 5), suggesting that Ca^2+^-activated Cl^-^ channels may be responsible for CFTR-independent PGE_2_-stimulated Cl^-^ secretion in ferret trachea.

**Fig 3:**
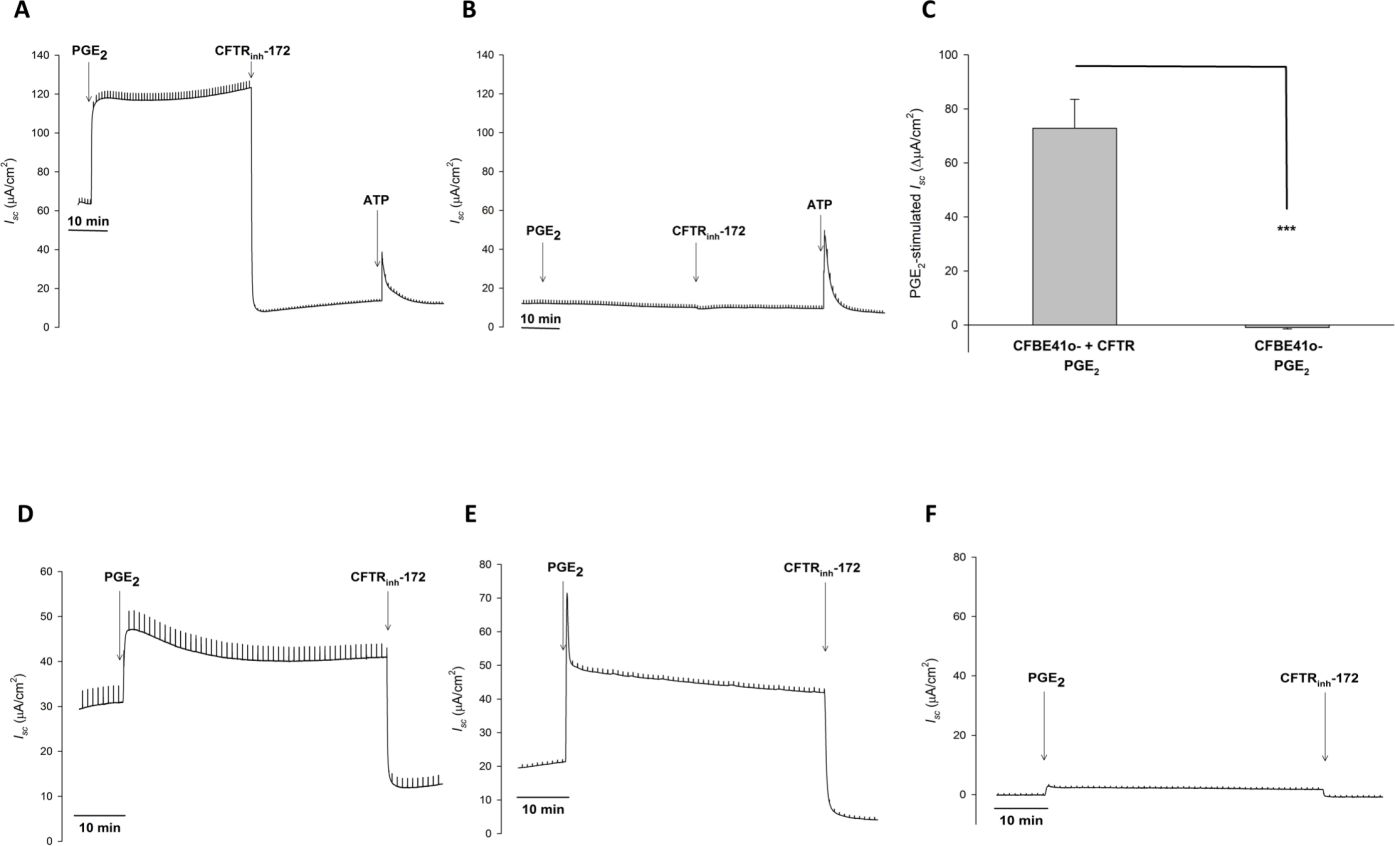
In human bronchial epithelial cells, PGE_2_ stimulated Cl^-^ secretion is completely CFTR dependent. **A.** Representative *I*_*sc*_ trace with vertical deflections indicating the change in *I*_*sc*_ after a 1 mV pulse was applied (every 1 minute). Bronchial epithelial cells were exposed to serosal to mucosal Cl^-^ gradient with equivalent bilateral HCO_3_^-^. PGE_2_ (1 μM, serosal) was added to HBE41 WT cells after a baseline period of ≥ 10 minutes, with CFTR_inh_-172 (20 μM, mucosal) added afterwards. To verify cell viability, ATP (500 μM, mucosal) was added. **B.** Representative *I*_*sc*_ trace from a similar experiment with CFBE41 CF cells. **C.** Change in PGE_2_-stimulated *I*_*sc*_ (mean ± SEM, n ≥ 4) in CFBE41 WT and CF cells. Asterisks denote significance by Student’s t-test (***, P < 0.001). Mean percent inhibition compared to CFBE41 WT noted. **D-F.** PGE_2_ stimulated Cl^-^ secretion in 16HBE14o- cells (D), primary cultures of human bronchial epithelial cells (E), and primary cultures from CF nasal polyp extract (F). Experiments were performed in the same manner as Fig 3A and representative *I*_*sc*_ traces are shown. N ≥ 3 experiments were performed for each set of cells with similar responses.

Since airway fluid is composed of secretions from both surface epithelial cells and submucosal glands, we next examined the individual contributions from cell culture models of bronchial epithelial cells and serous gland cells. We first examined PGE_2_-stimulated *I*_*sc*_ in CFBE41o- with transfected wildtype CFTR (CFBE41 WT) and with transfected F508del CFTR (CFBE41 CF) as models of surface epithelial cells. In the presence of amiloride (10 μM, mucosal), PGE_2_ (1 μM, serosal) stimulated a rapid and significant increase in *I*_*sc*_ over baseline in CFBE41 WT cells (47.84 ± 12.02 *vs*. 120.67 ± 4.75 μA/cm^2^, P < 0.01, n = 4). This response was completely abolished with CFTR_inh_-172 (20 μM, mucosal) (-41.47 ± 11.98 ΔμA/cm^2^ from baseline, n = 4). Given the magnitude of this inhibition, to ensure cells were still viable, ATP (500 μM, mucosal) was added after CFTR_inh_-172. ATP produced a rapid and transient increase in *I*_*sc*_ (Fig 3A). Similar experiments were performed with CFBE41 CF cells, which have little to no CFTR activity. In these cells PGE_2_ (1 μM, serosal) failed to stimulate *I*_*sc*_ (6.79 ± 1.69 *vs*. 5.84 ± 1.20 μA/cm^2^, P > 0.05, n = 5). CFTR_inh_-172 (20 μM, mucosal) had no effect (PGE_2_: 5.84 ± 1.20 *vs*. CFTR_inh_-172: 5.47 ± 1.39 μA/cm^2^, P > 0.05, n = 5), but ATP (500 μM, mucosal) did stimulate an increase in *I*_*sc*_ (Fig 3B). Thus, in bronchial epithelial cells, PGE_2_-stimulates transepithelial Cl^-^ secretion that is entirely CFTR-dependent (Fig 3C). Similar experiments were performed in the normal bronchial epithelial cell line 16HBE14o-, primary human bronchial epithelial cultures, and nasal cultures from CF patients, with similar responses to that in CFBE41 WT and CF cells (Fig 3D–3F), confirming that this was not a cell line-specific phenomenon.

**Fig 4:**
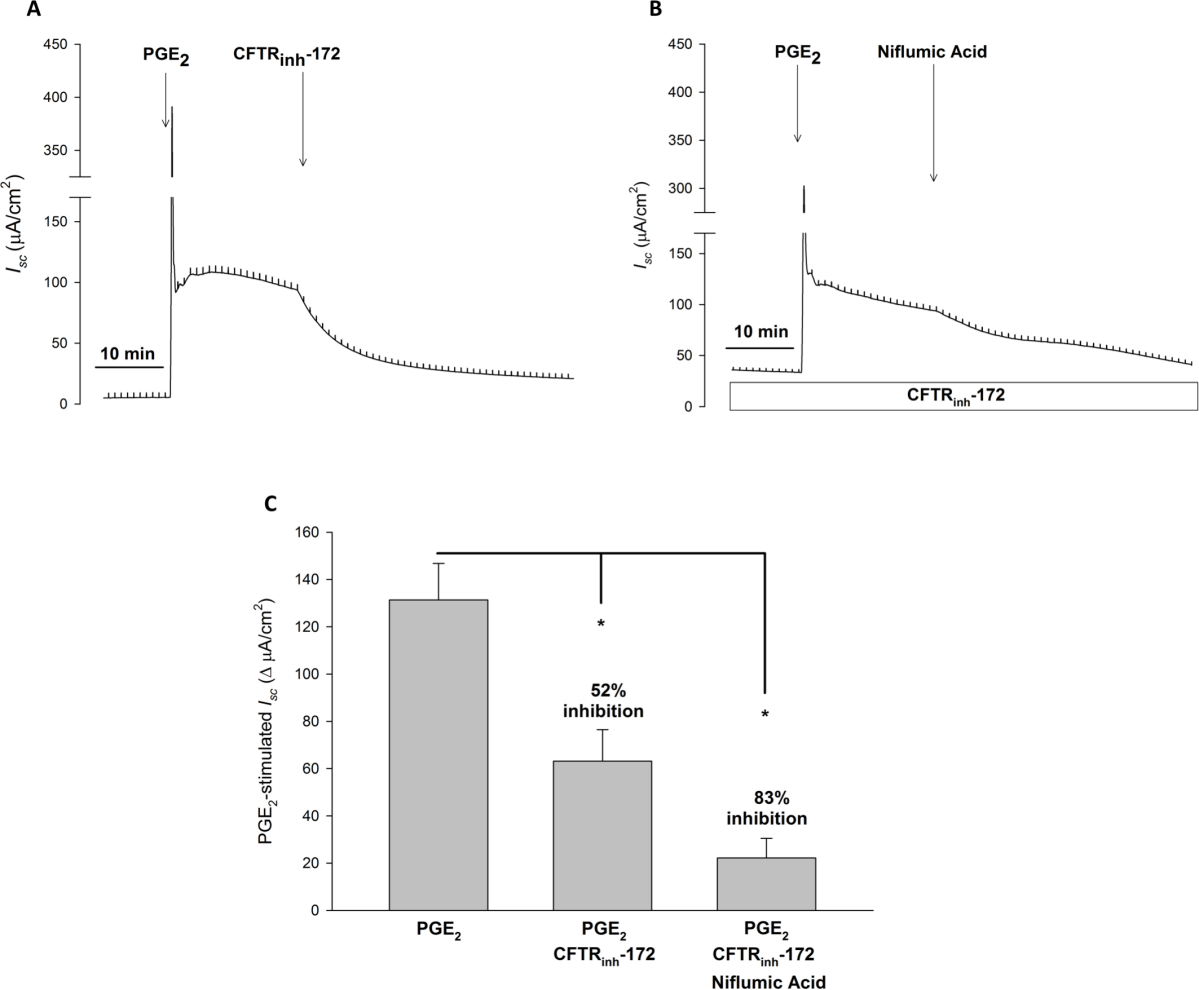
In Calu-3 cells, PGE_2_ stimulated Cl^-^ secretion is mediated by CFTR and Ca^2+^-activated Cl^-^ channels. **A.** Representative *I*_*sc*_ trace with vertical deflections indicating the change in *I*_*sc*_ after a 1 mV pulse was applied (every 1 minute). Calu-3 cells were exposed to serosal to mucosal Cl^-^ gradient with equivalent bilateral HCO_3_^-^. PGE_2_ (1 μM, serosal) was added to Calu-3 cells after a baseline period of ≥ 10 minutes, with CFTR_inh_-172 (20 μM, mucosal) added after 30 minutes. **B.** Representative *I*_*sc*_ trace of Calu-3 cells incubated in CFTR_inh_-172 (20 μM, mucosal) for at least 30 minutes prior to PGE_2_ (1 μM, serosal) stimulation. After 30 minutes, niflumic acid (100 μM, mucosal) was added. **C.** Change in PGE_2_-stimulated *I*_*sc*_ (mean ± SEM, n ≥ 4) in Calu-3 cells, with comparisons between no inhibition, CFTR inhibition, or CFTR and Ca^2+^-activated Cl^-^ inhibition. Asterisks denote significance by Student’s t-test (*, P < 0.05). Mean percent inhibition compared to PGE_2_ stimulation alone noted.

To examine PGE_2_-stimulated Cl^-^ secretion in serous gland cells, we used the Calu-3 cell line as a model. Experiments were performed in a similar manner as those done with bronchial epithelial cells. In the presence of amiloride (10 μM, mucosal), PGE_2_ (1 μM, serosal) stimulated a rapid and large transient increase in *I*_*sc*_, followed by a sustained significant increase in *I*_*sc*_ over baseline (17.61 ± 4.67 *vs*. 148.95 ± 18.51 μA/cm^2^, P < 0.001, n = 8). Subsequent addition of CFTR_inh_-172 (20 μM, mucosal) caused a robust, but incomplete, inhibition of PGE_2_-stimulated *I*_*sc*_ (PGE_2_: 148.95 ± 18.51 *vs*. CFTR_inh_-172: 53.90 ± 11.70 μA/cm^2^, P < 0.001, n = 8) (Fig 4A and 4C). Subsequent addition of bumetanide (10 μM, serosal), nearly eliminated the remaining PGE_2_-stimulated *I*_*sc*_ (8.15 ± 3.31 ΔμA/cm^2^ from baseline, n = 8), inhibiting PGE_2_-stimulated current by 94 ± 2%. Given the residual Cl^-^ current not inhibited by CFTR_inh_-172, we performed similar experiments to that done in ferret trachea and examined if NFA could inhibit this bumetanide-sensitive current. In the presence of CFTR_inh_-172 (20 μM, mucosal), NFA significantly inhibited PGE_2_-stimulated *I*_*sc*_ (PGE_2_+CFTR_inh_-172: 79.37 ± 16.57 *vs*. NFA: 38.40 ± 9.68 μA/cm^2^, P < 0.05, n = 4) (Fig 4B). Thus, similar to bronchial epithelial cells, PGE_2_ stimulates Cl^-^ secretion in Calu-3 cells, however, in contrast to bronchial epithelial cells, this current is not completely CFTR-dependent. Similar to what is seen in ferret trachea, CFTR-independent PGE_2_-stimulated Cl^-^ secretion is predominantly NFA-sensitive (Fig 4C).

### HCO_3_^-^ secretion

**Fig 5:**
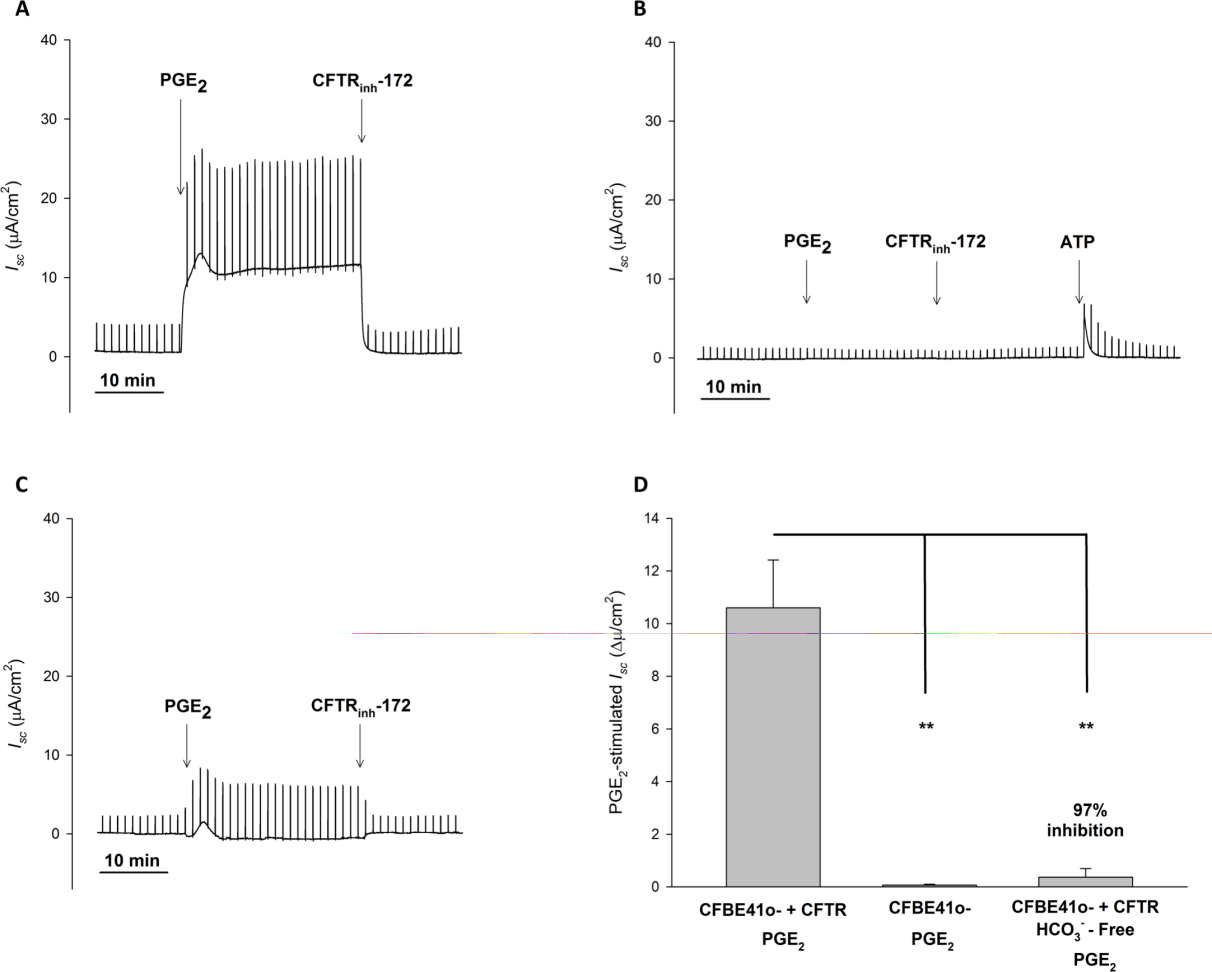
In CFBE41 cells, PGE_2_ stimulated HCO_3_^-^ secretion is completely CFTR dependent. **A.** Representative *I*_*sc*_ trace with vertical deflections indicating the change in *I*_*sc*_ after a 1 mV pulse was applied (every 1 minute). CFBE41 WT cells were exposed to serosal to mucosal HCO_3_^-^ gradient with equivalent bilateral Cl^-^. PGE_2_ (1 μM, serosal) was added to CFBE41 WT cells after a baseline period of ≥ 10 minutes, with CFTR_inh_-172 (20 μM, mucosal) added afterwards. **B.** Representative *I*_*sc*_ trace from a similar experiment with CFBE41 CF cells. To verify cell viability, ATP (500 μM, mucosal) was added. **C.** Representative *I*_*sc*_ trace from a similar experiment as Fig 5A with CFBE41 WT cells, except experiments were performed in HCO_3_^-^-free conditions. **D.** Change in PGE_2_-stimulated *I*_*sc*_ (mean ± SEM, n = 3) in CFBE41 WT and CF cells in HCO_3_^-^ containing and HCO_3_^-^-free conditions. Asterisks denote significance by Student’s t-test (**, P < 0.01). Mean percent inhibition compared to CFBE41 WT noted.

Having evaluated the effect of PGE_2_ on airway Cl^-^ secretion, we next sought to determine if PGE_2_ also stimulates airway HCO_3_^-^ secretion. To do so, we used the same human bronchial epithelial (CFBE41 WT and CF) and serous gland (Calu-3) cell models, and measured PGE_2_-stimulated *I*_*sc*_ with a serosal to mucosal HCO_3_^-^ gradient and symmetrical Cl^-^, in the presence of amiloride (10 μM, mucosal). In this configuration, PGE_2_ (1 μM, serosal) stimulated a significant increase in *I*_*sc*_ over baseline in CFBE41 WT cells (0.53 ± 0.05 *vs*. 11.13 ± 1.87 μA/cm^2^, P < 0.05, n = 3). Addition of CFTR_inh_-172 (20 μM, mucosal) abolished this response with *I*_*sc*_ returning to baseline levels (8.79 ± 2.003 *vs*. 0.57 ± 0.08 μA/cm^2^, P < 0.05, n = 3) (Fig 5A). PGE_2_ (1 μM, serosal) failed to stimulate *I*_*sc*_ in CFBE41 CF cells (Fig 5B), further supporting that the PGE_2_-stimulated HCO_3_^-^ conductance in bronchial epithelial cells relies on CFTR. Since this set-up contains both Cl^-^ and HCO_3_^-^ anions that can contribute to *I*_*sc*_, we next examined if PGE_2_-stimulated increases in *I*_*sc*_ were from HCO_3_^-^ or Cl^-^. To do so we performed identical experiments in HCO_3_^-^ free conditions with acetazolamide (300 μM, serosal) and 100% O_2_ mucosal gassing. In HCO_3_^-^ free conditions, PGE_2_ failed to increase *I*_*sc*_ above baseline (0.26 ± 0.15 *vs*. 0.65 ± 0.27 μA/cm^2^, P > 0.05, n = 3) (Fig 5C). These studies indicate that in CFBE41 WT cells, PGE_2_ stimulates HCO_3_^-^ transport that, similar to Cl^-^ transport in these cells, is entirely CFTR-dependent (Fig 5D).

**Fig 6:**
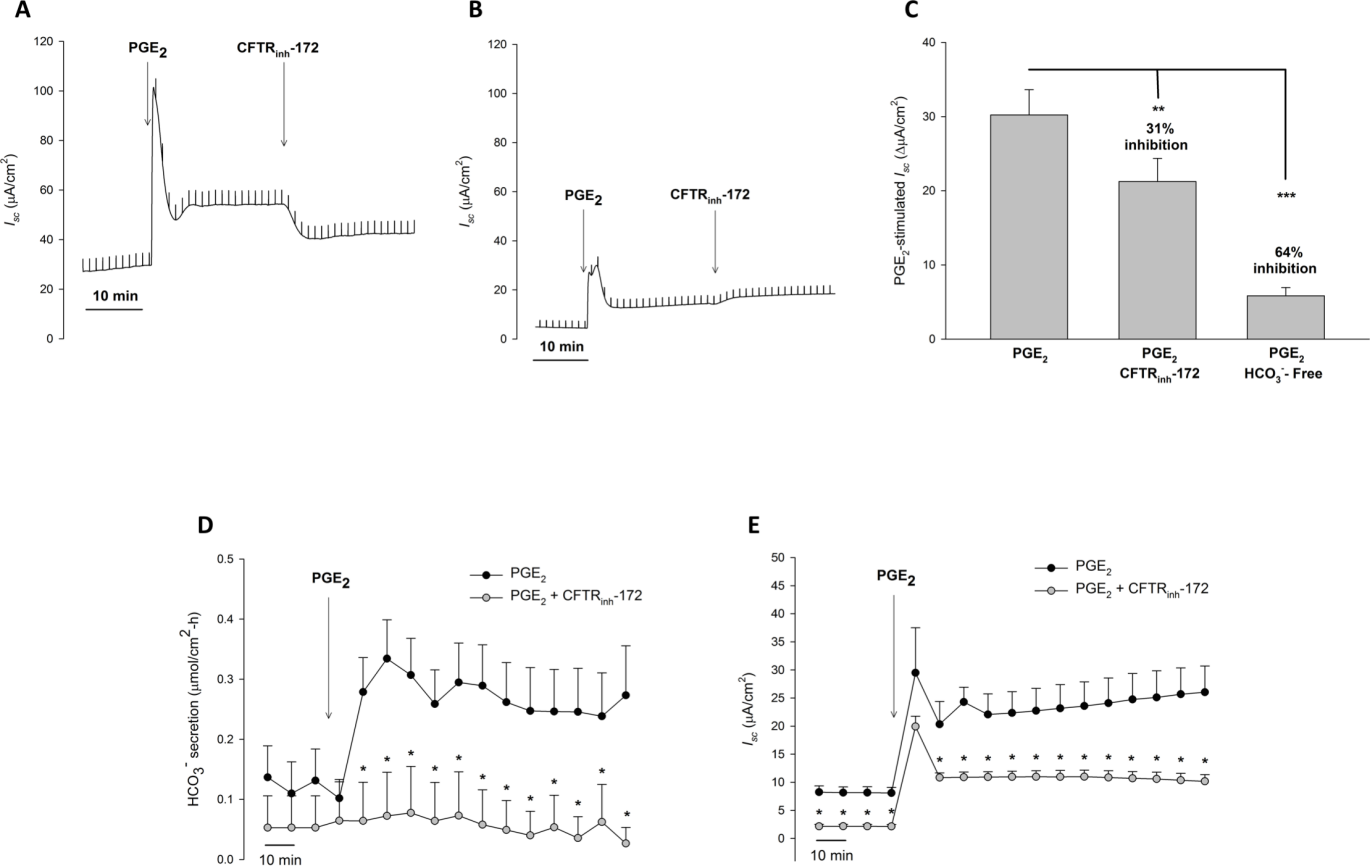
In Calu-3 cells, PGE_2_ stimulated HCO_3_^-^ secretion is completely CFTR dependent. **A.** Representative *I*_*sc*_ trace with vertical deflections indicating the change in *I*_*sc*_ after a 1 mV pulse was applied (every 1 minute). Calu-3 cells were exposed to serosal to mucosal HCO_3_^-^ gradient with equivalent bilateral Cl^-^. PGE_2_ (1 μM, serosal) was added to Calu-3 cells after a baseline period of ≥ 10 minutes, with CFTR_inh_-172 (20 μM, mucosal) added 30 minutes after. **B.** Representative *I*_*sc*_ trace from a similar experiment with Calu-3 cells in HCO_3_^-^-free conditions. **C.** Change in PGE_2_-stimulated *I*_*sc*_ (mean ± SEM, n = 3) in Calu-3 cells, with comparisons between no inhibition, CFTR inhibition, and HCO_3_^-^-free conditions. Asterisks denote significance by Student’s t-test (**, P < 0.01, ***, P < 0.001). Mean percent inhibition compared to Calu-3 cells under control conditions. **D.** Timecourse of HCO_3_^-^ secretion measured by pH-stat. The serosal solution was bathed with 95% O_2_/5% CO_2_ (similar to experiments in A-C), but the mucosal solution was bathed with 100% O_2_ to prevent base formation from carbonhic anhydrase conversion of CO_2_. Calu-3 cells were incubated in DMSO (5 μL; 1:1000 with bath; n = 10) or CFTR_inh_-172 (20 μM, mucosal; n = 6) for 30–60 minutes prior to PGE_2_ stimulation (1 μM, serosal). Circles represent means with bars indicating SEM. Asterisks represent P < 0.05 by ANOVA. **E.** Timecourse of *I*_*sc*_ measured by pH-stat measured simultaneously as pH-stat. Circles represent means with bars indicating SEM. Asterisks represent P < 0.05 by ANOVA.

**Fig 7:**
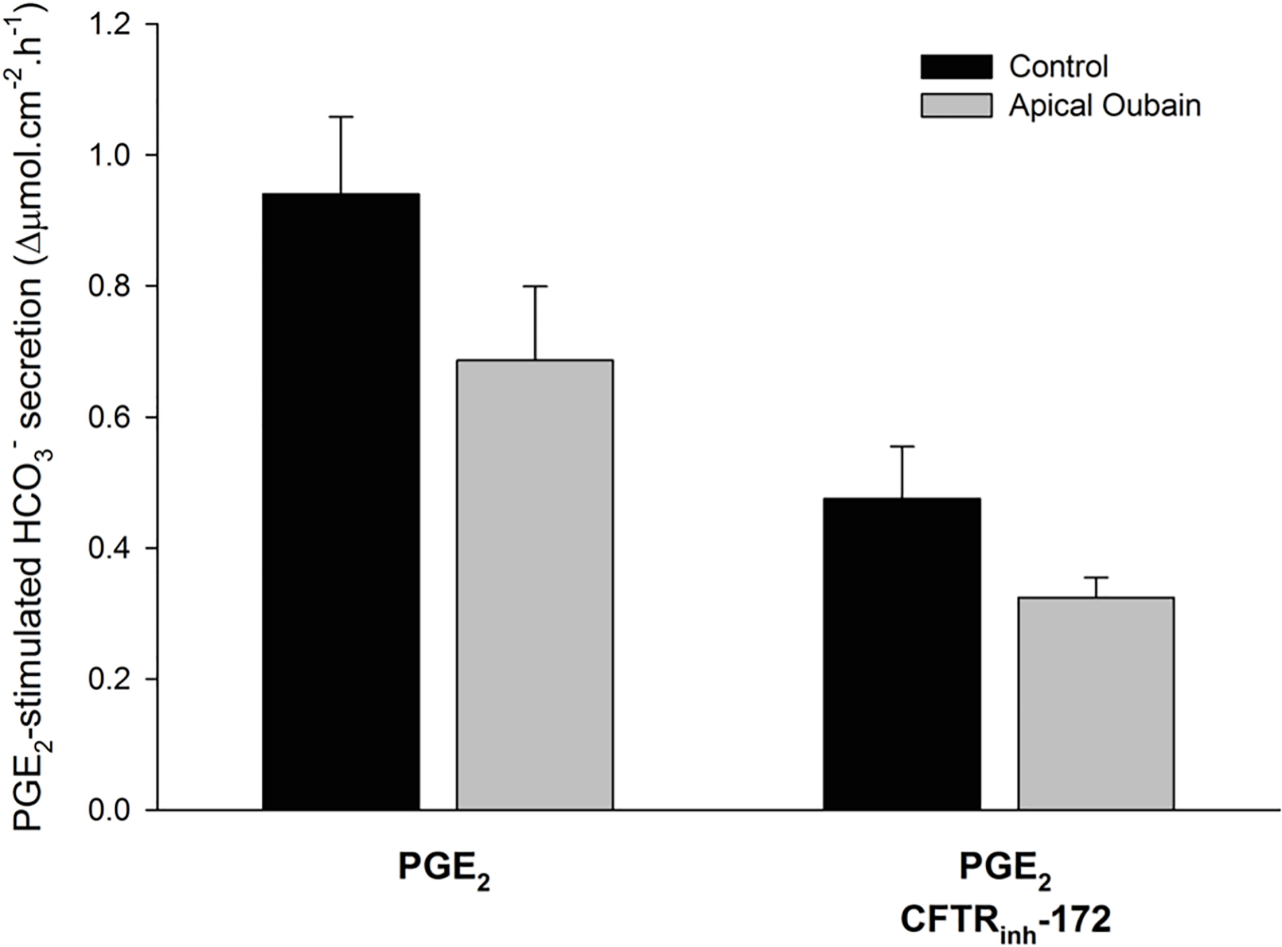
In Calu-3 cells, PGE_2_ stimulated HCO_3_^-^ secretion is not affected by apical oubain, an inhibitor of the non-gastric H^+^/K^+^ ATPase. Experiments were performed to determine the potential role of ATP12A in measured PGE_2_-stimulated HCO_3_^-^ secretion in normal and CF conditions. Calu-3 experiments were performed similar to that in Fig 6, with the exception that an additional set of experiments were done with oubain (10 μM, mucosal) pre-treatment for ≥ 40 minutes prior to PGE_2_ stimulation. Bars represent change in PGE_2_-stimulated *I*_*sc*_ (mean ± SEM, n ≥ 5) in Calu-3 cells. Statistical comparisons were done between PGE_2_ with and without oubain and PGE_2_ with CFTR inhibition with and without oubain. No statistical difference was noted in either case (P > 0.05 by Student’s t-test).

We next examined PGE_2_-stimulated HCO_3_^-^ secretion in Calu-3 cells. We first performed *I*_*sc*_ measurements in Ussing chambers, similar to that done with CFBE41 cells. Under these circumstances PGE_2_ caused a large, transient increase in *I*_*sc*_, followed by a sustained significant increase in *I*_*sc*_ (21.00 ± 2.30 *vs*. 51.22 ± 2.43 μA/cm^2^, P < 0.001, n = 7), which was markedly decreased (31%), but not completely eliminated by CFTR inhibition with CFTR_inh_-172 (51.22 ± 2.43 *vs*. 42.24 ± 1.53 μA/cm^2^, P < 0.01, n = 7) (Fig 6A and 6C). When repeating these experiments in HCO_3_^-^- free conditions, there remained a residual anion current stimulated by PGE_2_ (51.22 ± 2.43 *vs*. 42.24 ± 1.53 μA/cm^2^, P < 0.01, n = 7) that was resistant to CFTR_inh_-172 (Fig 6B and 6C). Given our prior findings suggesting that PGE_2_ can stimulate a Ca^2+^-activated Cl^-^ channel current in Calu-3 cells, we employed the pH-stat technique to measure HCO_3_^-^ secretion in a more direct manner. With this method, voltage clamp and pH-stat were simultaneously measured with Calu-3 cells exposed to symmetrical Cl^-^ and a serosal to mucosal HCO_3_^-^ gradient with mucosal O_2_ gassing to prevent the generation of apical base from gassed CO_2_. To mitigate any potential influences of drugs on apical pH, these experiments were performed with DMSO (5 μL; 1:1000 with bath) or CFTR_inh_-172 (20 μM, mucosal) added prior to PGE_2_ stimulation. With this method, PGE_2_-stimulated a significant increase in HCO_3_^-^ secretion in control conditions (n = 10, P > 0.05). In contrast, CFTR inhibition ameliorated this response (n = 6, P < 0.05) (Fig 6D). Similar to prior experiments, PGE_2_-stimulated a significant increase in *I*_*sc*_, that was partially inhibited with CFTR_inh_-172 (P < 0.05) (Fig 6E). To ensure that activation of apical non-gastric H^+^/K^+^ ATPase did not cause falsely low HCO_3_^-^ secretory rates, we performed similar experiments with or without apical ouabain (10 μM). Oubain did not significantly alter PGE_2_-stimulated HCO_3_^-^ secretion, with or without CFTR_inh_-172 (n ≥ 5, P > 0.05) (Fig 7).

## Discussion

### PGE_2_ and CF airway disease

Cystic fibrosis affects approximately 30,000 people in the U.S., with an estimated annual mean healthcare cost of approximately 1.5 Billion U.S. dollars [25]. The majority of healthcare costs, morbidity, and mortality associated with CF are attributed to pulmonary infections and their associated complications. Amongst the inflammatory milieu of the infected airways, PGE_2_ is abundantly produced by epithelia and infiltrating inflammatory cells, and is found in bronchioalveolar lavage fluid, sputum, and airway epithelium [7–10]. Lack of functional CFTR may tilt the balance into excessive PGE_2_ production, leading to a positive proinflammatory loop of NF-kβ (nuclear factor-kappa beta) and CREB (cAMP response element binding protein) activation, causing an upregulation of cyclooxygenase-2 (COX-2) and increased PGE_2_ production [26]. The overall result being an exaggerated inflammatory condition. Ibuprofen, which can be a useful therapeutic agent in CF [27], may help tip the balance of PGE_2_ back to appropriate levels. In addition to promoting inflammation, PGE_2_ also helps resolve inflammation by stimulating Cl^-^, HCO_3_^-^, and mucin secretion [11]. Bronchotracheal MCC is integral to the innate mucosal defense against microbial insults and is regulated by coordinated efforts between transepithelial Cl^-^ secretion and submucosal gland mucus secretion. In this study, we have shown for the first time that PGE_2_ stimulates MCC in ferret trachea. We also showed that CFTR inhibition causes a significant decrease (~50%) in the initial phase of MCC, indicating that PGE_2_-stimulated MCC in CF patients may be impaired. The inability of CFTR_inh_-172 to have a more substantial impact on MCC may be related to the activation of non-CFTR Cl^-^ channels, supported by the ability of niflumic acid to further inhibit CFTR_inh_-172-independent *I*_*sc*_, or relative insensitivity of ferret CFTR channels to CFTR_inh_-172 [28]. The former hypothesis is supported by our Calu-3 data, which also showed sensitivity to both CFTR_inh_-172 and niflumic acid. Activation of TMEM16A channels increases ciliary beat frequency and ASL height, both of which would increase MCC [29]. Likewise, Joo *et al*. found that in the ferret trachea forskolin-, but not carbachol-stimulated MCC was inhibited by CFTR_inh_-172 [30]. Thus, we speculate that the residual PGE_2_-induced MCC during CFTR inhibition may be due to Ca^2+^-activated Cl^-^ channel activity.

### Chloride secretion

We are not the first group to examine PGE_2_-stimulated anion transport in the airway, however, we have undertaken the most comprehensive examination of PGE_2_-stimulated Cl^-^ and HCO_3_^-^ secretion to date. Cullen and Widdicombe *et al*. showed that PGE_2_ increases *I*_*sc*_ in canine and human trachea [15, 31], while Cowley showed the same in Calu-3 cells [32]. In the latter study, PGE_2_-stimulated *I*_*sc*_ was inhibited 87% by pre-incubation with DPC (diphenylamine-2-carboxylate), suggesting significant CFTR-dependence [32]. Before newer generation CFTR inhibitors, DPC was commonly used to inhibit CFTR. However, DPC is not specific for CFTR and can inhibit other Cl^-^ channels [33]. In our bronchial epithelial experiments, we found that CFTR_inh_-172 was a potent and complete inhibitor of PGE_2_-stimulated Cl^-^ secretion. As such, we speculate that the CFTR_inh_-172-independent *I*_*sc*_ observed in ferret trachea and Calu-3 cells is due to non-CFTR Cl^-^ channels. Widdicombe et al. observed small increases in PGE_2_-stimulated *I*_*sc*_ in CF human trachea that was unresponsive to isoproterenol [31]. With the ability of niflumic acid to inhibit our observed residual current, we hypothesize that Ca^2+^-activated Cl^-^ channels account for the CFTR_inh_-172-independent *I*_*sc*_ in ferret trachea and Calu-3 cells. Shamsuddin *et al* found that complete inhibition of PGE_2_-stimulated *I*_*sc*_ in small porcine airways required both CFTR and Ca^2+^-activated channel inhibition (GlyH-101 and niflumic acid, respectively) [16].

**Fig 8:**
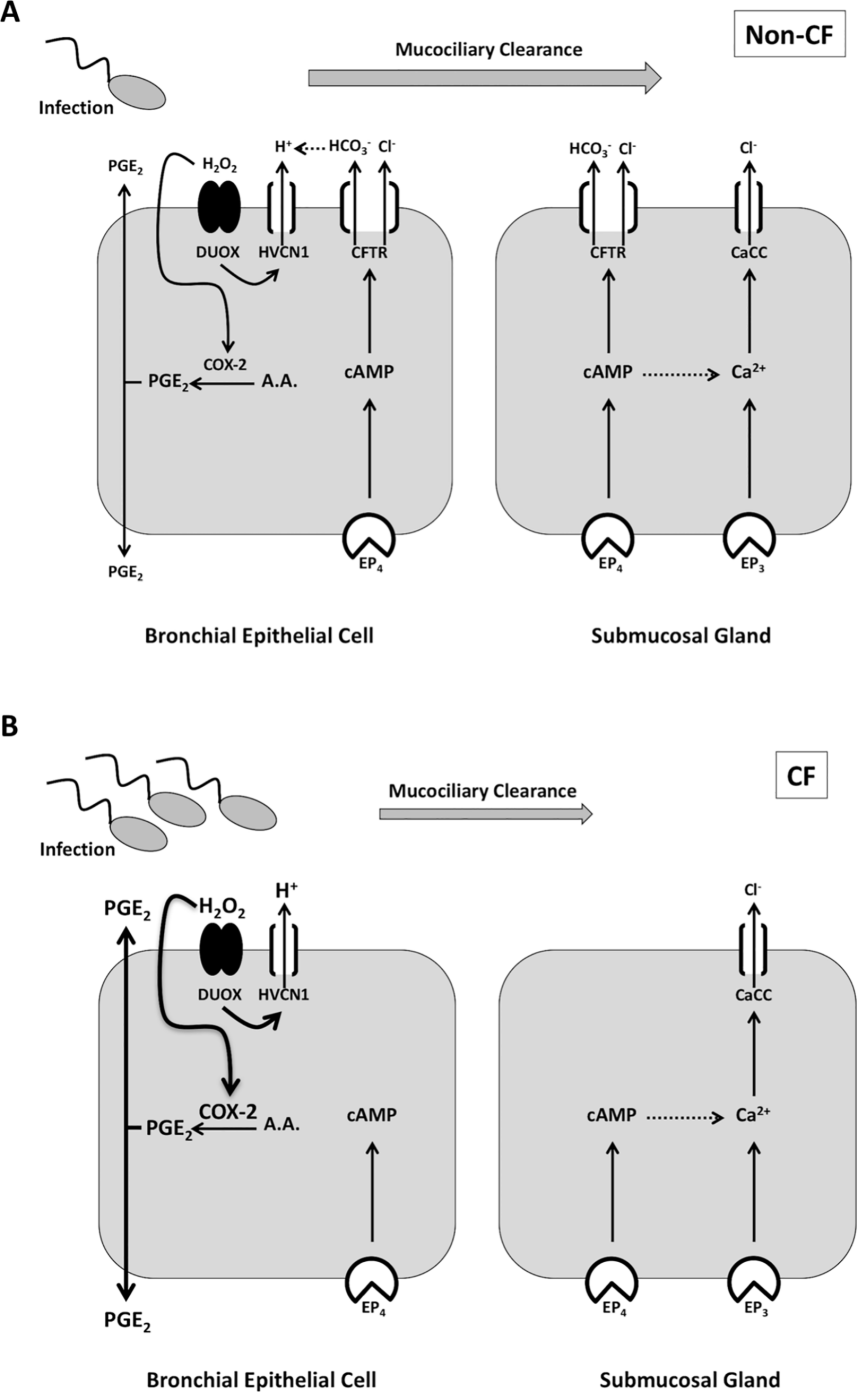
Simplified working model of PGE_2_-stimulated Cl^-^ and HCO_3_^-^ secretion and mucociliary clearance in non-CF and CF airway. **A.** In the airway, microbial infections cause an increase in PGE_2_ through release from infiltrating inflammatory cells (not pictured) and production by airway epithelia *via* COX-2 activation. H_2_O_2_ produced by DUOX activates COX-2 and HVCN1 channels provide the H^+^ shunt from H_2_O_2_ production. PGE_2_ exits the cell and activates PGE_2_ (EP) receptors. In the current study we did not examine specific EP receptor involvement, however, we propose that EP_4_ is the predominant mediator of serosal PGE_2_ stimulation in bronchial epithelial cells. Submucosal gland cells may also utilize the EP_3_ receptor, or Ca^2+^-activated Cl^-^ channels (CaCC) may get activated *via* EP_4_-mediated cAMP-Ca^2+^ crosstalk. In bronchial epithelial cells, PGE_2_ stimulates Cl^-^ and HCO_3_^-^ secretion *via* CFTR, whereas in submucosal glands, both CFTR and CaCC are activated. Cl^-^ and HCO_3_^-^ secretion will then influence airway pH, mucus properties, hydration, and ultimately, mucociliary clearance. **B.** In CF airway, lack of CFTR-dependent Cl^-^ and HCO_3_^-^ secretion in bronchial epithelial cells, coupled with no HCO_3_^-^ secretion and decreased Cl^-^ secretion from submucosal glands, leads to an acidic airway pH, thick-adherent mucus, and decreased mucociliary clearance. This results in increased microbial infection and rampant inflammation, in part by increased PGE_2_ production.

We found differential responses to PGE_2_-stimulated Cl^-^ secretion between bronchial epithelial cell lines and submucosal gland cell lines. In WT CFBE41 and other bronchial epithelial cell lines (including primary culture), PGE_2_-stimulated Cl^-^ secretion required CFTR. However, Calu-3 cells appear to utilize both CFTR and Ca^2+^-activated Cl^-^ channels. Ferret trachea showed similar responses to Calu-3 cells, likely due to the presence of submucosal glands. The difference in responses is unlikely to be due to a lack of Ca^2+^-activated Cl^-^ channels in our bronchial epithelial cell cultures since apical ATP stimulated Cl^-^ current in both WT and CFBE41 cells. It is possible that there is differential PGE_2_ receptor expression between the two cell types. Four different receptors for PGE_2_ have been described (EP_1_-EP_4_), with all four being expressed in Calu-3 cells. EP_1_ and EP_2_ receptors are located at the apical membrane, while EP_3_ and EP_4_ receptors are located at both the apical and basolateral membranes [17]. In normal human tracheobronchial epithelial (NHTBE) cells, EP_1_-EP_4_ mRNA are present [34], however, to our knowledge, there are no published reports examining EP receptor membrane expression in CFBE41o- cells or other surface airway epithelial cells. EP_1_ and EP_3_ signaling increases intracellular Ca^2+^, while EP_3_ can also stimulate inositol triphosphate (IP_3_). EP_2_ and EP_4_ increase cAMP, while EP_4_ also stimulates PI3K [35]. In the duodenum, PGE_2_ stimulates HCO_3_^-^ secretion *via* cAMP, Ca^2+^, and PI3K through EP_3_ and EP_4_ receptors [12]. In Calu-3 cells, Joy *et al*. found that CFTR-dependent PGE_2_-stimulated iodide efflux was mediated by EP_4_ [17]. This leads one to hypothesize that CFTR-dependent Cl^-^ secretion in bronchial epithelial cells and Calu-3 cells may be mediated by EP_4_, whereas Ca^2+^-activated Cl^-^ secretion in Calu-3 cells may occur through EP_3_ activation. Ongoing studies examining the EP receptor membrane distribution in bronchial epithelial cells may shed light on this hypothesis (Fig 8). It may also be possible that there is different intracellular signaling machinery in bronchial epithelial cells and Calu-3 cells, leading to cAMP and Ca^2+^ crosstalk in Calu-3, but not, bronchial epithelial cells. In mouse inner medullary collecting duct cells, PGE_2_ stimulated CFTR_inh_-172- and flufenamic acid-sensitive *I*_*sc*_ exclusively through EP_4_ receptors. Inhibition of IP_3_ receptors with 2-APB (aminoethoxyldiphenyl borate) blocked PGE_2_-stimulated *I*_*sc*_ by nearly 80%, with complete inhibition of the Ca^2+^-activated Cl^-^ current. [36]. Lee *et al*. have described cAMP-dependent activation of IP_3_-dependent Ca^2+^ release in submucosal glands and Joo *et al*. have recently shown that low dose forskolin and carbachol can generate a synergistic *I*_*sc*_ and MCC response in ferret trachea [30, 37]. Intracellular increases in cAMP may bind to Epac (exchange protein directly activated by cAMP), catalyzing the generation of IP_3_ by phospholipase C, resulting in release of intracellular Ca^2+^ stores and eventual Ca^2+-^activated Cl^-^ channel activation [38, 39]. Namkung *et al*. also showed that elevations in intracellular Ca^2+^ can also lead to activation of Ca^2+^/calmodulin-sensitive adenylyl cyclase 1, further illustrating the possible bidirectional activation of cAMP and Ca^2+^ signaling pathways [40]. In the intestine, lubiprostone, a prostaglandin derivative, increases the trafficking of EP_4_ and CFTR to the membrane, which would be anticipated to increase ion transport [41]. It is unclear if a similar mechanism occurs in bronchial epithelial cells or submucosal glands when exposed to PGE_2_. Ongoing research into the receptor dependence of PGE_2_ stimulation in bronchial epithelial cells and submucosal glands, and the intracellular signaling and trafficking involved in Ca^2+^-activated Cl^-^ channel activation may lead to new ideas on how to coopt this mechanism as a therapeutic target in CF.

### Bicarbonate secretion

In recent years there has been increased focus on airway HCO_3_^-^ transport, as it has become apparent that defective ASL formation cannot be accounted for by altered Cl^-^ and Na^+^ alone [42–44]. The role of HCO_3_^-^ secretion in MCC is less clear than that of Na^+^ or Cl^-^. Jeong *et al*. found that HCO_3_^-^ removal did not decrease MCC in a statistically significant manner [2]. However, HCO_3_^-^ removal impairs submucosal gland secretion and mucus detachment, both of which would negatively affect MCC [45, 46]. Airway HCO_3_^-^ secretion has been confirmed previously in both surface epithelial cells and submucosal glands [44, 47]. Shamsuddin *et al*. showed that PGE_2_ stimulates HCO_3_^-^ transport in porcine small airways [16], but we are the first to examine PGE_2_-stimulated HCO_3_^-^ secretion in both bronchial epithelial and Calu-3 cells. In addition to CFTR, HCO_3_^-^ conductance can be regulated through increased HCO_3_^-^ uptake by the Na:HCO_3_^-^ cotransporter, basolateral Cl^-^/HCO_3_^-^ exchange and/or intracellular HCO_3_^-^ generation by carbonic anhydrase. In the current study, we did not examine the individual roles of these processes in generating HCO_3_^-^ substrate, however, we did show that PGE_2_-stimulated HCO_3_^-^ secretion in the airway requires CFTR. This may occur through direct HCO_3_^-^ transport through CFTR and/or apical recycling of CFTR-mediated Cl^-^ secretion through apical Cl^-^/HCO_3_^-^ anion exchangers [19, 48]. In the duodenum, apical HCO_3_^-^ conductance through Cl^-^/HCO_3_^-^ exchangers can occur in an electroneutral manner, independent of CFTR [13]. Our pH-stat data did not show any electroneutral HCO_3_^-^ secretion, indicating that, in contrast to the duodenum [13], CFTR-independent Cl^-^/HCO_3_^-^ exchange is likely not involved in PGE_2_-stimulated HCO_3_^-^ secretion in Calu-3 cells.

It remains unclear whether the effects of HCO_3_^-^ transport loss in CF are due to acidic pH or HCO_3_^-^ itself. To address this question, Tang *et al*. examined CF porcine ASL viscosity at variable HCO_3_^-^ concentrations and pH values. In their experiments, ASL viscosity was primarily affected by pH, not HCO_3_^-^ concentration itself [3]. In addition to HCO_3_^-^ transport, H^+^ secretion also helps to regulate ASL pH. Lung epithelium contains DUOX NADPH oxidase, which produces H_2_O_2_ for release during pulmonary microbial infections. Schwarzer *et al*. showed that Zn^2+^-sensitive HVCN1 channels shunt H^+^ generated during DUOX NADPH oxidase reactions out of the cell [49]. This would serve to lower ASL pH. Interestingly, H_2_O_2_ causes autocrine release of PGE_2_ and stimulates CFTR-dependent increases in *I*_*sc*_ [50]. Thus, one might hypothesize that H_2_O_2_ release during acute pulmonary infections may increase PGE_2_-stimulated Cl^-^ secretion to increase MCC and increase HCO_3_^-^ secretion to limit the negative effect of H_2_O_2_-induced H^+^ secretion on ASL pH (Fig 8). Iovannisci *et al*. also showed that HVCN1 H^+^ channels are activated by ASL pH, being closed at resting ASL pH of 6.85 and become active as the pH alkalinizes above that [23]. In our *I*_*sc*_ experiments we did not examine H^+^ secretion specifically. These experiments were done at pH 7.4 so in theory the HVCN1 H^+^ channel could be active. The lack of *I*_*sc*_ response to PGE_2_ in bronchial epithelial cells without CFTR or with HCO_3_^-^ removal suggests that HVCN1 H^+^ channels were either not activated by PGE_2_ or they play an insignificant role. Likewise, our pH-stat experiments were performed at a set point of pH 6.9 to ensure that HVCN1 activation did not mask HCO_3_^-^ secretion. Another potential contributor to apical H^+^ transport and ASL pH in porcine and human airway epithelium is ATP12A (the α subunit of non-gastric H^+^/K^+^ ATPase). Shah *et al*. showed that at pH 7.0, in CF human and pig airway epithelia, cAMP stimulated a decrease in ASL pH, which was inhibited by apical oubain or siRNA against ATP12A [51]. Thus, it has been proposed that in the absence of CFTR, increases in cAMP may lead to ATP12A activation and acidify the ASL. In our bronchial epithelial studies, similar to the reasons stated above, we did not examine ATP12A channel activation. However, we did perform a set of experiments in Calu-3 cells with pH-stat where we applied apical oubain to determine if this unmasked a change in PGE_2_-stimulated apical pH. We found no change with or without ouabain, leading us to conclude that in Calu-3 cells, under our experimental conditions, PGE_2_ does not activate ATP12A channels. Altogether, we do not have any evidence that PGE_2_ activates HVCN1 or ATP12A channels, although specific studies examining PGE_2_-stimulated H^+^ secretion in different pH environments may be warranted. What we can say is that PGE_2_ does stimulate HCO_3_^-^ secretion in bronchial epithelial cells and submucosal glands and we propose that together with Cl^-^ secretion, this contributes to increasing MCC and microbial removal during infection.

### Conclusions

In summary, we have shown that PGE_2_, an inflammatory mediator produced during CF pulmonary exacerbations, is involved in bronchotracheal MCC and the stimulation of Cl^-^ and HCO_3_^-^ secretion from bronchial epithelial cells and submucosal glands. Absence of CFTR activity in bronchial epithelial cells leads to a total loss of both Cl^-^ and HCO_3_^-^ secretion. In submucosal glands, HCO_3_^-^ secretion is absent, yet some niflumic acid-sensitive Cl^-^ secretion remains, suggesting involvement of Ca^2+^-activated Cl^-^ secretion. This residual anion current may mitigate some of the deleterious effects of CFTR loss on MCC. These studies provide further information on the role of CFTR in maintaining airway health and provide additional insight into CF airway pathology. Further work to understand the mechanism whereby PGE_2_ may stimulate Ca^2+^-activated Cl^-^ channels and MCC in CF, may help identify new therapeutic targets that may assist in the normalization of airway ion transport and clearance of pulmonary microbial insults.
